# *N*-(2-Chloro­phen­yl)-3-methyl­benzamide

**DOI:** 10.1107/S160053681000992X

**Published:** 2010-03-20

**Authors:** Vinola Zeena Rodrigues, Miroslav Tokarčík, B. Thimme Gowda, Jozef Kožíšek

**Affiliations:** a Department of Chemistry, Mangalore University, Mangalagangotri 574 199, Mangalore, India; b Faculty of Chemical and Food Technology, Slovak Technical University, Radlinského 9, SK-812 37 Bratislava, Slovak Republic

## Abstract

In the title compound, C_14_H_12_ClNO, the N—H bond is *anti* to the carbonyl bond and the two aromatic rings make a dihedral angle of 5.4 (2)°. In the crystal, inter­molecular N—H⋯O hydrogen bonds connect the mol­ecules into chains running along the *b* axis. The chains are inter­connected through short Cl⋯Cl contacts [3.279 (1) Å].

## Related literature

For the preparation of the compound, see: Gowda *et al.* (2003 ▶). For related structures, see: Bowes *et al.* (2003 ▶); Gowda *et al.* (2008**a* ▶,b* ▶).
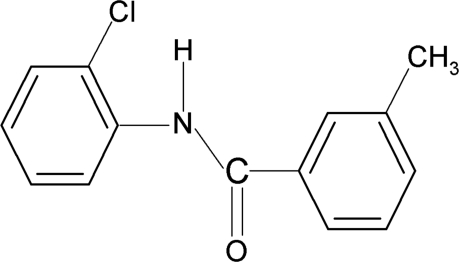


## Experimental

### 

#### Crystal data

C_14_H_12_ClNO
*M* *_r_* = 245.7
Monoclinic, 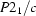

*a* = 9.9972 (3) Å
*b* = 4.9124 (1) Å
*c* = 24.6662 (7) Å
β = 100.248 (3)°
*V* = 1192.04 (5) Å^3^
*Z* = 4
Mo *K*α radiation
μ = 0.30 mm^−1^
*T* = 295 K
0.55 × 0.35 × 0.08 mm

#### Data collection

Oxford Diffraction Xcalibur diffractometer with a Ruby Gemini detector
Absorption correction: analytical (*CrysAlis PRO*; Oxford Diffraction, 2009 ▶) *T*_min_ = 0.897, *T*_max_ = 0.978
25420 measured reflections
2119 independent reflections
1884 reflections with *I* > 2σ(*I*)
*R*_int_ = 0.050

#### Refinement

*R*[*F* ^2^ > 2σ(*F* ^2^)] = 0.041
*wR*(*F* ^2^) = 0.108
*S* = 1.05
2119 reflections
157 parameters
H-atom parameters constrained
Δρ_max_ = 0.22 e Å^−3^
Δρ_min_ = −0.22 e Å^−3^

### 

Data collection: *CrysAlis PRO* (Oxford Diffraction, 2009 ▶); cell refinement: *CrysAlis PRO*; data reduction: *CrysAlis PRO*; program(s) used to solve structure: *SHELXS97* (Sheldrick, 2008 ▶); program(s) used to refine structure: *SHELXL97* (Sheldrick, 2008 ▶); molecular graphics: *ORTEP-3* (Farrugia, 1997 ▶) and *DIAMOND* (Brandenburg, 2002 ▶); software used to prepare material for publication: *SHELXL97*, *PLATON* (Spek, 2009 ▶) and *WinGX* (Farrugia, 1999 ▶).

## Supplementary Material

Crystal structure: contains datablocks global. DOI: 10.1107/S160053681000992X/bt5219sup1.cif

Structure factors: contains datablocks I. DOI: 10.1107/S160053681000992X/bt5219Isup2.hkl

Additional supplementary materials:  crystallographic information; 3D view; checkCIF report

## Figures and Tables

**Table 1:** Hydrogen-bond geometry (Å, °)

*D*—H⋯*A*	*D*—H	H⋯*A*	*D*⋯*A*	*D*—H⋯*A*
N1—H1*N*⋯O1^i^	0.86	2.16	2.936 (2)	151

Symmetry code: (i) 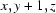.

**Fig. 1.**
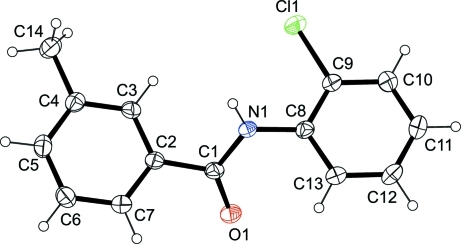
Molecular structure of (I) showing the atom labelling scheme. Displacement ellipsoids are drawn at the 30% probability level and H atoms are represented as small spheres of arbitrary radii.

**Fig. 2.**
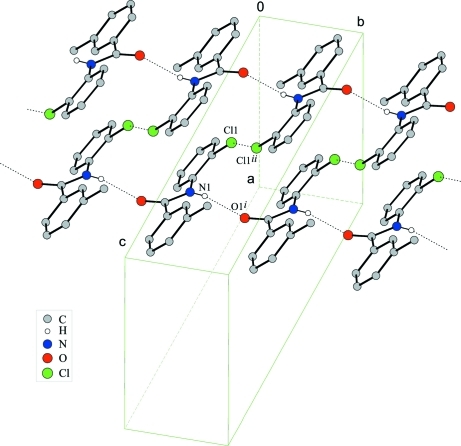
Part of crystal structure of (I) showing molecular chains running along the b-axis and interconnected through the Cl—Cl contacts. Hydrogen bonds and short Cl—Cl contacts are shown as dashed lines. H atoms not involved in hydrogen bonding were omitted. Symmetry codes: (i) x, 1+y, z; (ii) -x, 1-y, 1-z.

## supplementary crystallographic information

### Comment

As part of a study of the substituent effects on the crystal structures of benzanilides (Gowda *et al.*, 2008*a,b*), the structure of *N*-(2-chlorophenyl)3-methylbenzamide (I) has been determined. In the structure, the conformations of the N—H and C=O bonds are *anti* to each other (Fig. 1), similar to those observed in in *N*-(2-chlorophenyl)2-methylbenzamide (II), *N*-(2-chlorophenyl)benzamide (III)(Gowda *et al.*, 2008*b*), 3-methyl-*N*-(phenyl)benzamide (IV) (Gowda *et al.*, 2008*a*) and the parent benzanilide (Bowes *et al.*, 2003). Further, the conformation of the C=O bond in (I) is also *anti* to the *meta*- methyl substituent in the benzoyl ring, while the conformation of the N—H bond is *syn* to the *ortho*-Cl group in the aniline ring..

The central amide group –NH—C(=O)– is twisted by 35.6 (2)° and 37.9 (2)° out of the planes of the 3-methylphenyl and 2-chlorophenyl rings, respectively.

The dihedral angle between the two benzene rings is 5.4 (2)°, compared to the values of 7.4 (3)° in (II) and 22.2 (2)°) & 75.9 (1), in the molecules 1 and 2 of (IV), respectively.

The packing diagram of molecules in (I) showing the intermolecular N–H···O hydrogen bonds (Table 1) involved in the formation of molecular chains running along the *b*-axis is shown in Fig. 2. The chains are interconnected through short Cl···Cl contacts of 3.279 (1) Å.

### Experimental

The title compound was prepared according to the literature method (Gowda *et al.*, 2003). The purity of the compound was checked by determining its melting point. It was characterized by recording its infrared and NMR spectra. Single crystals of the title compound used in X-ray diffraction studies were obtained from a slow evaporation of its ethanolic solution at room temperature.

### Refinement

All hydrogen atoms were positioned with idealized geometry using a riding model with C–H = 0.93 Å or 0.96 Å and N–H = 0.86 Å. The *U*_iso_(H) values were set at 1.2*U*_eq_(C_aromatic_, N) or 1.5*U*_eq_(C_methyl_). The C14-methyl group exhibits orientational disorder in the positions of H atoms. The two sets of methyl hydrogen atoms were refined with occupancies of 0.66 (3) and 0.34 (3).

### Crystal data

**Table:**

C_14_H_12_ClNO	*F*(000) = 512
*M**_r_* = 245.7	*D*_x_ = 1.369 Mg m^−^^3^
Monoclinic, *P*2_1_/*c*	Mo *K*α radiation, λ = 0.71073 Å
Hall symbol: -P 2ybc	Cell parameters from 16100 reflections
*a* = 9.9972 (3) Å	θ = 2.1–29.5°
*b* = 4.9124 (1) Å	µ = 0.30 mm^−^^1^
*c* = 24.6662 (7) Å	*T* = 295 K
β = 100.248 (3)°	Block, colourless
*V* = 1192.04 (5) Å^3^	0.55 × 0.35 × 0.08 mm
*Z* = 4	

### Data collection

**Table:**

Oxford Diffraction Xcalibur diffractometer with a Ruby Gemini detector	2119 independent reflections
graphite	1884 reflections with *I* > 2σ(*I*)
Detector resolution: 10.434 pixels mm^-1^	*R*_int_ = 0.050
ω scans	θ_max_ = 25.1°, θ_min_ = 2.1°
Absorption correction: analytical (*CrysAlis PRO*; Oxford Diffraction, 2009)	*h* = −11→11
*T*_min_ = 0.897, *T*_max_ = 0.978	*k* = −5→5
25420 measured reflections	*l* = −29→29

### Refinement

**Table:**

Refinement on *F*^2^	Primary atom site location: structure-invariant direct methods
Least-squares matrix: full	Secondary atom site location: difference Fourier map
*R*[*F*^2^ > 2σ(*F*^2^)] = 0.041	Hydrogen site location: inferred from neighbouring sites
*wR*(*F*^2^) = 0.108	H-atom parameters constrained
*S* = 1.05	*w* = 1/[σ^2^(*F*_o_^2^) + (0.0534*P*)^2^ + 0.5999*P*] where *P* = (*F*_o_^2^ + 2*F*_c_^2^)/3
2119 reflections	(Δ/σ)_max_ < 0.001
157 parameters	Δρ_max_ = 0.22 e Å^−^^3^
0 restraints	Δρ_min_ = −0.22 e Å^−^^3^

### Special details

**Table:**

Geometry. All esds (except the esd in the dihedral angle between two l.s. planes) are estimated using the full covariance matrix. The cell esds are taken into account individually in the estimation of esds in distances, angles and torsion angles; correlations between esds in cell parameters are only used when they are defined by crystal symmetry. An approximate (isotropic) treatment of cell esds is used for estimating esds involving l.s. planes.
Refinement. Refinement of *F*^2^ against ALL reflections. The weighted *R*-factor wR and goodness of fit *S* are based on *F*^2^, conventional *R*-factors *R* are based on *F*, with *F* set to zero for negative *F*^2^. The threshold expression of *F*^2^ > σ(*F*^2^) is used only for calculating *R*-factors(gt) etc. and is not relevant to the choice of reflections for refinement. *R*-factors based on *F*^2^ are statistically about twice as large as those based on *F*, and *R*- factors based on ALL data will be even larger.

### Fractional atomic coordinates and isotropic or equivalent isotropic displacement parameters (Å^2^)

**Table:**

	*x*	*y*	*z*	*U*_iso_*/*U*_eq_	Occ. (<1)
C1	0.29838 (18)	−0.1699 (3)	0.57259 (7)	0.0361 (4)	
C2	0.28794 (18)	−0.0729 (3)	0.62913 (7)	0.0351 (4)	
C3	0.19358 (19)	0.1212 (4)	0.63886 (8)	0.0379 (4)	
H3	0.1363	0.1997	0.6091	0.045*	
C4	0.18309 (19)	0.2001 (4)	0.69191 (8)	0.0396 (4)	
C5	0.2713 (2)	0.0838 (4)	0.73564 (8)	0.0459 (5)	
H5	0.2666	0.1359	0.7715	0.055*	
C6	0.3659 (2)	−0.1087 (4)	0.72660 (8)	0.0479 (5)	
H6	0.4246	−0.1841	0.7563	0.057*	
C7	0.3737 (2)	−0.1891 (4)	0.67377 (8)	0.0425 (5)	
H7	0.4362	−0.3211	0.6679	0.051*	
C8	0.27931 (18)	−0.0271 (3)	0.47628 (7)	0.0349 (4)	
C9	0.18735 (19)	0.1059 (4)	0.43608 (8)	0.0385 (4)	
C10	0.1894 (2)	0.0693 (5)	0.38088 (8)	0.0523 (5)	
H10	0.1269	0.1598	0.3546	0.063*	
C11	0.2843 (3)	−0.1017 (5)	0.36470 (9)	0.0583 (6)	
H11	0.2861	−0.1271	0.3275	0.07*	
C12	0.3762 (2)	−0.2344 (5)	0.40389 (9)	0.0539 (5)	
H12	0.4401	−0.3504	0.393	0.065*	
C13	0.3748 (2)	−0.1975 (4)	0.45914 (8)	0.0437 (5)	
H13	0.4382	−0.2873	0.4852	0.052*	
C14	0.0792 (2)	0.4081 (4)	0.70160 (9)	0.0534 (5)	
H14A	0.0666	0.3986	0.7392	0.08*	0.66 (3)
H14B	−0.0056	0.3716	0.6776	0.08*	0.66 (3)
H14C	0.1103	0.5868	0.6941	0.08*	0.66 (3)
H14D	0.0476	0.506	0.6681	0.08*	0.34 (3)
H14E	0.1198	0.5331	0.7297	0.08*	0.34 (3)
H14F	0.0039	0.3179	0.7132	0.08*	0.34 (3)
N1	0.27660 (16)	0.0202 (3)	0.53244 (6)	0.0373 (4)	
H1N	0.2597	0.1834	0.5419	0.045*	
O1	0.32539 (16)	−0.4076 (3)	0.56431 (6)	0.0511 (4)	
Cl1	0.06537 (6)	0.31991 (12)	0.45519 (2)	0.0569 (2)	

### Atomic displacement parameters (Å^2^)

**Table:**

	*U*^11^	*U*^22^	*U*^33^	*U*^12^	*U*^13^	*U*^23^
C1	0.0421 (10)	0.0259 (9)	0.0396 (10)	0.0012 (7)	0.0058 (8)	0.0004 (7)
C2	0.0404 (10)	0.0263 (9)	0.0390 (10)	−0.0015 (7)	0.0081 (8)	0.0021 (7)
C3	0.0458 (10)	0.0287 (9)	0.0389 (10)	0.0016 (8)	0.0067 (8)	0.0041 (7)
C4	0.0465 (11)	0.0300 (9)	0.0447 (10)	−0.0042 (8)	0.0149 (8)	−0.0015 (8)
C5	0.0594 (13)	0.0444 (11)	0.0355 (10)	−0.0052 (9)	0.0129 (9)	−0.0041 (8)
C6	0.0527 (12)	0.0505 (12)	0.0384 (10)	0.0024 (10)	0.0025 (9)	0.0049 (9)
C7	0.0457 (11)	0.0378 (10)	0.0441 (11)	0.0067 (8)	0.0079 (8)	0.0029 (8)
C8	0.0412 (10)	0.0264 (9)	0.0381 (9)	−0.0006 (7)	0.0096 (7)	−0.0024 (7)
C9	0.0423 (10)	0.0330 (9)	0.0410 (10)	0.0054 (8)	0.0097 (8)	−0.0023 (8)
C10	0.0593 (13)	0.0581 (13)	0.0383 (10)	0.0138 (11)	0.0052 (9)	0.0003 (9)
C11	0.0753 (15)	0.0626 (14)	0.0405 (11)	0.0123 (12)	0.0195 (10)	−0.0065 (10)
C12	0.0595 (13)	0.0512 (12)	0.0556 (13)	0.0149 (10)	0.0227 (10)	−0.0088 (10)
C13	0.0469 (11)	0.0386 (10)	0.0464 (11)	0.0100 (9)	0.0099 (9)	−0.0013 (9)
C14	0.0628 (14)	0.0444 (11)	0.0571 (13)	0.0064 (10)	0.0222 (10)	−0.0039 (10)
N1	0.0514 (9)	0.0248 (7)	0.0361 (8)	0.0072 (7)	0.0091 (7)	−0.0016 (6)
O1	0.0801 (10)	0.0252 (7)	0.0494 (8)	0.0073 (6)	0.0152 (7)	−0.0007 (6)
Cl1	0.0570 (3)	0.0622 (4)	0.0510 (3)	0.0279 (3)	0.0085 (2)	−0.0044 (2)

### Geometric parameters (Å, °)

**Table:**

C1—O1	1.224 (2)	C9—C10	1.377 (3)
C1—N1	1.350 (2)	C9—Cl1	1.7375 (18)
C1—C2	1.495 (3)	C10—C11	1.378 (3)
C2—C7	1.392 (3)	C10—H10	0.93
C2—C3	1.392 (3)	C11—C12	1.374 (3)
C3—C4	1.386 (3)	C11—H11	0.93
C3—H3	0.93	C12—C13	1.377 (3)
C4—C5	1.389 (3)	C12—H12	0.93
C4—C14	1.507 (3)	C13—H13	0.93
C5—C6	1.384 (3)	C14—H14A	0.96
C5—H5	0.93	C14—H14B	0.96
C6—C7	1.377 (3)	C14—H14C	0.96
C6—H6	0.93	C14—H14D	0.96
C7—H7	0.93	C14—H14E	0.96
C8—C9	1.390 (3)	C14—H14F	0.96
C8—C13	1.391 (3)	N1—H1N	0.86
C8—N1	1.410 (2)		
			
O1—C1—N1	123.31 (17)	C8—C9—Cl1	119.86 (14)
O1—C1—C2	120.91 (16)	C9—C10—C11	119.87 (19)
N1—C1—C2	115.78 (15)	C9—C10—H10	120.1
C7—C2—C3	119.03 (17)	C11—C10—H10	120.1
C7—C2—C1	118.18 (16)	C12—C11—C10	119.63 (19)
C3—C2—C1	122.76 (16)	C12—C11—H11	120.2
C4—C3—C2	121.43 (17)	C10—C11—H11	120.2
C4—C3—H3	119.3	C11—C12—C13	120.66 (19)
C2—C3—H3	119.3	C11—C12—H12	119.7
C3—C4—C5	118.30 (17)	C13—C12—H12	119.7
C3—C4—C14	120.62 (18)	C12—C13—C8	120.58 (19)
C5—C4—C14	121.08 (18)	C12—C13—H13	119.7
C6—C5—C4	120.91 (18)	C8—C13—H13	119.7
C6—C5—H5	119.5	C4—C14—H14A	109.5
C4—C5—H5	119.5	C4—C14—H14B	109.5
C7—C6—C5	120.24 (18)	C4—C14—H14C	109.5
C7—C6—H6	119.9	C4—C14—H14D	109.5
C5—C6—H6	119.9	C4—C14—H14E	109.5
C6—C7—C2	120.07 (18)	H14D—C14—H14E	109.5
C6—C7—H7	120	C4—C14—H14F	109.5
C2—C7—H7	120	H14D—C14—H14F	109.5
C9—C8—C13	117.94 (17)	H14E—C14—H14F	109.5
C9—C8—N1	119.86 (16)	C1—N1—C8	125.30 (15)
C13—C8—N1	122.16 (17)	C1—N1—H1N	117.3
C10—C9—C8	121.32 (17)	C8—N1—H1N	117.3
C10—C9—Cl1	118.82 (15)		
			
O1—C1—C2—C7	34.3 (3)	N1—C8—C9—C10	178.40 (18)
N1—C1—C2—C7	−145.58 (18)	C13—C8—C9—Cl1	179.69 (14)
O1—C1—C2—C3	−143.6 (2)	N1—C8—C9—Cl1	−2.4 (2)
N1—C1—C2—C3	36.5 (2)	C8—C9—C10—C11	−0.1 (3)
C7—C2—C3—C4	−0.2 (3)	Cl1—C9—C10—C11	−179.31 (18)
C1—C2—C3—C4	177.67 (17)	C9—C10—C11—C12	0.0 (4)
C2—C3—C4—C5	1.1 (3)	C10—C11—C12—C13	−0.2 (4)
C2—C3—C4—C14	−179.28 (17)	C11—C12—C13—C8	0.6 (3)
C3—C4—C5—C6	−0.9 (3)	C9—C8—C13—C12	−0.8 (3)
C14—C4—C5—C6	179.54 (19)	N1—C8—C13—C12	−178.60 (18)
C4—C5—C6—C7	−0.3 (3)	O1—C1—N1—C8	1.2 (3)
C5—C6—C7—C2	1.3 (3)	C2—C1—N1—C8	−178.98 (16)
C3—C2—C7—C6	−1.0 (3)	C9—C8—N1—C1	142.39 (19)
C1—C2—C7—C6	−178.99 (18)	C13—C8—N1—C1	−39.8 (3)
C13—C8—C9—C10	0.5 (3)		

### Hydrogen-bond geometry (Å, °)

**Table:**

*D*—H···*A*	*D*—H	H···*A*	*D*···*A*	*D*—H···*A*
N1—H1N···O1^i^	0.86	2.16	2.936 (2)	151

Symmetry codes: (i) *x*, *y*+1, *z*.
